# MAPk Activation Modulates Permeability of Isolated Rat Alveolar Epithelial Cell Monolayers Following Cyclic Stretch

**DOI:** 10.1371/journal.pone.0010385

**Published:** 2010-04-28

**Authors:** Taylor S. Cohen, Gladys Gray Lawrence, Amit Khasgiwala, Susan S. Margulies

**Affiliations:** Department of Bioengineering, University of Pennsylvania, Philadelphia, Pennsylvania, United States of America; University of Giessen Lung Center, Germany

## Abstract

We cultured (5 days) rat alveolar epithelial cells to investigate the role of mitogen-activated protein kinase (MAPk) signaling in ventilator induced epithelial barrier dysfunction. Cells were stretched to a magnitude of 12% or 37% change in surface area at a rate of 0.25 Hz with and without pretreatment with either the JNK inhibitor SP600125 or the ERK inhibitor U0126. Following stretch (0, 10, 30, or 60 min), MAPk phosphorylation was examined, monolayer permeability to the uncharged tracer carboxyfluorescein measured (0, 10, 60 min of stretch), and occludin expression determined (0 and 60 min of stretch). Stretch to 12%, previously shown not to increase monolayer permeability, did not alter phosphorylation of any MAPk or occludin expression at any time point. Following stretch to 37%, phosphorylation of JNK, ERK, and p38 was significantly higher by 10 minutes than in unstretched monolayers. Phosphorylation of JNK and p38 subsided as stretch continued, and by 30 minutes returned to unstretched levels. Phosphorylation of ERK remained significantly elevated compared to unstretched levels at all stretch durations. Epithelial permeability increased significantly by 10 minutes of stretch compared to unstretched controls, with further significant increases by 60 minutes. Inhibition with U0126 and SP600125 prevented stretch-induced phosphorylation increases of ERK and JNK, respectively, however neither prevented increases in permeability following 10 minutes. Separately, inhibition of JNK or ERK prevented subsequent additional permeability increases as stretch continued to 60 minute time points. Inhibition of JNK, not ERK, prevented loss of occludin, and minimized loss of cell-cell contact following 60 minutes of stretch. These data suggest that stretch-induced JNK signaling modulates epithelial permeability through regulation tight junction protein expression, and is a potential target for clinical treatments during mechanical ventilation.

## Introduction

Ventilator induced lung injury (VILI) is a significant complication to mechanically ventilated patients, as 5–15% of these patients will develop VILI, and of those approximately 35–60% of these cases are fatal [1], [2], [3]. Past work, both in our lab and elsewhere, has shown that the large magnitudes to which the lung is stretched during ventilation is a primary cause of VILI [4], [5], [6], [7]. Furthermore, an important clinical trial by the ARDSnet corroborates these studies by showing improved mortality rates in patients ventilated with a lower tidal volume (6 ml/kg compared to 12 ml/kg) [8].

Recent work in our lab has shown that 1 hour of cyclic (0.25 Hz) stretch of cultured alveolar epithelial monolayers to a peak magnitude corresponding to 100% TLC increases trancellular permeability [4], [9]. In this communication we explore the role of mitogen activated protein kinase (MAPk) signaling in stretch-induced barrier dysfunction.

The MAPk family of serine-threonine protein kinases is a highly conserved family consisting of the three primary members; extracellular signal-related kinases (ERK), c-Jun amino-terminal kinases (JNK), and p38. MAPks play an active signaling role following multiple stimuli, some of which include irradiation, osmotic stress, inflammation, growth factors, and mechanical loading [10], [11], [12], [13]. Downstream from the MAPks are a range of transcription factors which control multiple cellular responses such as inflammation, proliferation, and apoptosis [14], [15], [16].

In the lung, MAPk signaling pathway activation has been observed following mechanical ventilation. MAPk activation was also shown to increase with increased magnitude of ventilation and to correlate with increases in mortality, circulatory failure, and pulmonary edema [17], [18]. While numerous studies have investigated the role of MAPks in cellular function, there is a paucity of data examining their role in regulating alveolar epithelial permeability in the presence of mechanical stretch.

In this communication, we examined phosphorylation of ERK, JNK, and p38 following the application of cyclic stretch to a magnitude previously shown to produce barrier dysfunction. Using inhibitors to block specific pathways, we investigated functional relationships of these pathways in transducing stretch to barrier permeability. We concluded that the JNK and ERK signaling pathways negatively regulate barrier function following stretch, however JNK, not ERK, is associated with observed stretch induced reductions in occludin expression.

## Methods

### Cell Isolation

Primary rat alveolar epithelial type II cells were isolated from pathogen-free male Sprague-Dawley rats (250–350 g) via an elastase digestion technique adopted from Dobbs *et. al.* [19]. After isolation, cells were re-suspended in a solution of minimum essential medium (MEM), 10% fetal bovine serum (FBS), 0.4 µl/ml Gentamicin, and 1 µl/ml Amphotericin B (Life Technologies, Rockville, MD) and seeded (1*10^6^ cells/cm^2^) on either an elastic substrate or permeable co-polyester membranes (Mylan Technologies, Burlington, VT), both of which were mounted in custom-built polysulfone wells. The permeable membrane was previously corona discharge-treated, and coated with poly-L-lysine (0.5 µg/cm^2^; Sigma, St. Louis, MO) and fibronectin (10 µg/cm^2^; Boehringer Mannheim Biochemicals, Indianapolis, IN) to improve cell adhesion. All wells were incubated for a period of 5 days, at which point we have demonstrated they lose alveolar epithelial type II phenotypic markers and take on type I phenotypic markers [20], [21]. The media was changed daily.

### Inhibition of MAP Kinases

Following 5 days in culture, wells were washed with Delbecco's Modified Eagle's Medium (DMEM) and serum deprived for 2 hours, then treated for 1 hour with 1.5 mls of DMEM containing either SP600125 (20 or 35 µM) or U0126 (10 or 20 µM) to inhibit JNK or ERK phosphorylation respectively (Cell Signaling, Danvers, MA). DMSO and U0124 (20 µM) were used as a negative control in viability and dose response studies for the inhibitors SP60125 and U0126 respectively. DMSO was used as a vehicle control for both inhibitors in all permeability studies.

### Cell Monolayer Stretching Protocol

Monolayers were stretched in the presence of DMEM, DMEM and a MAPk inhibitor, or DMEM and vehicle control (DMSO). Wells were mounted in a custom-made stretch device capable of applying a uniform and equibiaxial cyclic stretch to the cells at a magnitude of 12% or 37% ΔSA and frequency of 0.25 Hz [22]. This stretch magnitude corresponds to the strain experienced by the alveolar epithelium *in vivo* at an inflation from functional residual capacity to 70% or 100% total lung capacity (TLC), or tidal volume 12ml/kg or 30 ml/kg in the rat with no end-expiratory pressure (PEEP). Three sets of stretch experiments were conducted as follows. First, the appropriate dose of each inhibitor and vehicle control was determined by stretching a group of 8 total wells from 2 rat isolations for 10 minutes, as this was the stretch duration found to produce peak MAPk phosphorylation in pilot studies. The dose which best inhibited stretch induced activation of the targeted MAPk while not being toxic to the cells was chosen. Dose response for SP600125 was evaluated at concentrations of 20 and 35 µM, and for U0126 at 10 and 20 µM. Second, stretch-induced MAPk activation was determined by stretching a group of 16 wells (with DMEM) from 4 separate isolations for four stretch durations (0, 10, 30, and 60 minutes). Finally, the effect of MAPk inhibition on epithelial paracellular permeability was determined by stretching groups of 4 wells (with DMEM and the inhibitor) from 3 isolations for a total of 12 wells per inhibitor at three stretch durations (0, 10, and 60 minutes). The temperature of the stretch device was maintained at 37°C.

### Cell Viability Measurement

A subset of wells was used to determine cellular toxicity of the inhibitors SP600125 and U0126 at the maximum concentration used in the dose response study, 35 µM and 20 µM respectively. We used 2 wells from each of the following unstretched groups: DMSO vehicle, SP600125, U0126, and each of the following stretched (37% ΔSA, 0.25 Hz) groups: DMSO vehicle, SP600125, U0124, and U0126. Each well was treated, immediately prior to stretch, with Ethidium Homodimer-1 (7.5 µM), which stains the nuclei of dead cells, and Calcein AM (16 µM) to label live cells (Molecular Probes, Eugene, OR). Following the 60 minute stretch period, wells were imaged in three locations (20× objective) using a fluorescent microscope (Nikon TE-300, Melville NY), the number of dead and live adherent cells were determined, and toxicity was calculated as the ratio of dead cells divided by the total number of cells (dead plus live cells). Average toxicity was determined from the mean of the 6 images (3 regions from each of 2 wells) for each treatment and stretch condition. Comparisons were made across treatments, and between stretched and unstretched fields.

### Cell Monolayer Tracer Transport Measurements

After a 10 or 60 minute stretch period, wells (N = 12 per group) were removed from the stretch device and mounted into Ussing chambers within approximately 2 minutes. The apical to basal paracellular permeability of the monolayers was assessed using methods similar to those previously described [4]. Briefly, both apical and basal sides of the Ussing chamber were filled with 500 µl of Ringer's solution. A 360 kDa fluorescent molecular tracer, Carboxyfluorescein, in a 10% wt/vol Ringer's solution was added to the apical chamber (Sigma, St. Louis, MO). The system was allowed to incubate for 2 hours while the tracer passively diffused from the apical to basal chamber through the cell monolayer. Following the 2 hour period, the basal fluid was removed and the tracer concentration was assessed using a 96 well plate fluorimeter (GMI, Ramsey, MN), with an excitation filter of 485nm and emission filter of 538 nm.

We assumed diffusive transport of the tracer, with no sources or sinks, and used well-established equations governing conservation of mass [6], [23]. Transport followed first-order Fickian diffusion described by:
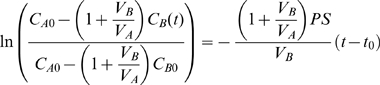
(1)where *C_A0_* = initial apical tracer concentration, *C_B0_* = initial basal tracer concentration, *C_B_(t)* = basal concentration at time *t*, *P* = paracellular permeability of the monolayer to a specific tracer (units = length/time), *S* = surface area over which permeability occurs. Since all variables but *P* are known, we were able to solve for permeability directly. Previously, we assessed permeability change in 15 minute intervals up to 2 hours post stretch, and reported no change in monolayer permeability over this time period [4].

### Immunoblot Analysis of MAPKs and Occludin

Rat alveolar epithelial cells (3 isolations of 4 wells per group) in primary culture were scraped from the silastic membrane in the presence of chilled radio-immunoprecipitation assay (RIPA) buffer containing 4.3 mM ethylenediaminetetraacetic acid (EDTA) and a cocktail of protease and phosphotase inhibitors, and placed on ice to minimize cellular activity. Equal protein lysates were run on SDS-polyacrylamide (10%) reducing gels, transferred onto polyvinylidene fluoride membranes (PVDF) and non-specific binding was blocked in tris buffered saline (TBS) containing 5% non-fat powdered milk and 0.05% Tween-20 at 4°C. The PVDF membranes were initially probed for phosphorylated MAP kinases or occludin then stripped and reprobed for total MAP kinases or actin (MAPk antibodies from Cell Signaling Technology, Beverly, MA, occludin and actin antibodies from Invitrogen, San Diego, CA). MAPk activity and occludin and actin concentration was calculated through densitometric analysis using Kodak 1D Image Analysis Software (Eastman Kodak, Rochester, NY). Band intensities for phospho-MAPks were normalized by respective total protein levels, and occludin was normalized to actin concentration. Stretched data was then normalized to the unstretched control lane on each blot for comparisons across blots.

### Tight Junction Protein Immunofluorescence

Following the stretch period (0 or 60 minutes), cells (2 isolations of 2 wells per group) were washed with phosphate-buffered saline (PBS), fixed for 15 min with 1.5% paraformaldehyde, washed again in PBS, and treated for 5 min with 0.1% Triton X-100 in PBS. Cells were blocked for 1 hour in 10% normal goat serum, then incubated overnight with either 2.0 µg/ml occludin or 1.0 µg/ml ZO-1 antibodies (Invitrogen). After washing in PBS, the cells were incubated for 2 hours in secondary antibody (Jackson Laboratories), mounted, and imaged (Nikon TE-300, 40× objective).

### Statistical Analysis

An initial analysis was used to determine effects of different doses of MAPk inhibitors SP600125 and U0126 on JNK and ERK phosphorylation compared to their vehicle controls DMSO and U0124, following a stretch duration of 10 min. Student's t-tests were conducted between inhibitor and vehicle control data for each MAPk to determine the effectiveness of the inhibitor. Significance was defined as p≤0.05.

To determine the effect of stretch duration on MAPk phosphorylation in cultured monolayers, separate analyses was performed for each kinase JNK, ERK, and p38, comparing across stretch durations (0, 10, 30, and 60 min) using a one-way ANOVA. Subsequently, post hoc Tukey tests were used to determine significant difference in kinase phosphorylation at individual time points [24] (JMP ver. 4, SAS Institute, Cary, NC).

To determine the effect of MAPk inhibition on epithelial permeability, separate analyses for each inhibitor and vehicle control combination were performed, comparing across stretch duration using a two-way ANOVA. Post hoc Tukey tests were used to analyze individual comparisons between time points for the same treatment or treatments at the same time point. Statistical significance was defined as p≤0.05.

To determine the effect of stretch and MAPk signaling on occludin concentration, occludin expression was compared across stretch (0 and 60 minutes) and treatment (DMSO, SP600125, and U0126) combinations using a two-way ANOVA. Post hoc Tukey tests were used for individual comparisons.

### Ethics Statement

All animal use was done in accordance with, and with the approval of, the IACUC in the Office of Regulatory Affairs of the University of Pennsylvania.

## Results

### Mechanical Deformation Results in Magnitude and Time Dependent MAPk Phosphorylation

**Figure 1:**
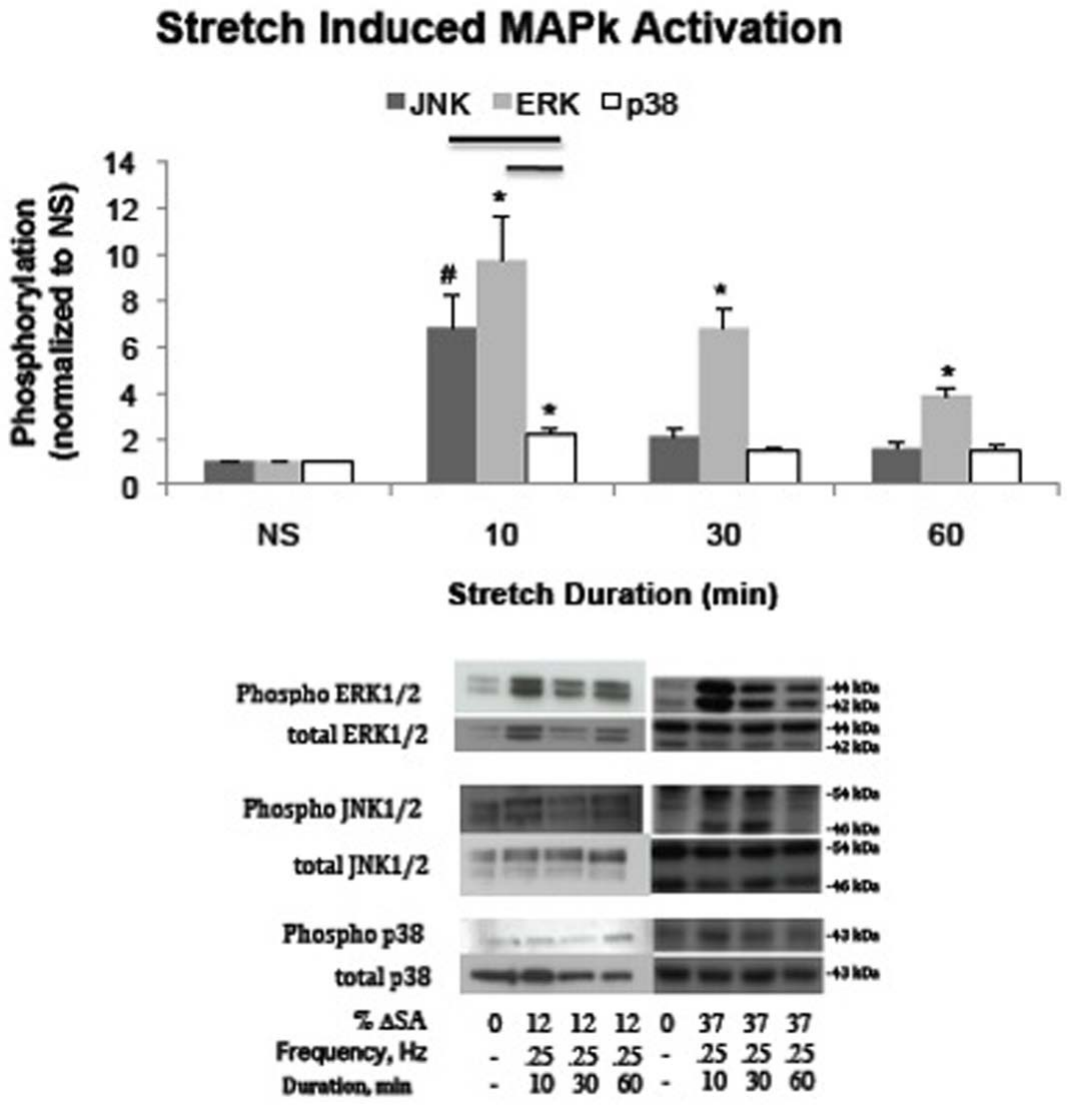
Stretch activation of MAPk signaling. Phosphorylation levels of JNK, ERK, and p38 MAPk following 10, 30, and 60 minutes of cyclic stretch at a rate of 0.25 Hz to a peak magnitude of 37% ΔSA normalized to unstretched controls. Phosphorylation levels of all MAPks significantly increased above unstretched levels (value of 1) following 10 minutes of stretch (*). Following 30 or 60 minutes of stretch, only ERK remained significantly elevated. JNK phosphorylation was significantly greater following 10 minutes than following 60 minutes (#). At the 10 minute time point, phosphorylation of JNK and ERK was significantly higher than phosphorylation of p38. Significance is defined as p<0.05. Representative western blots of phosphorylated and total MAPks following stretch to either 12% or 37% ΔSA are also shown. No change in phosphorylated protein normalized to total protein was observed at any stretch duration with a stretch magnitude of 12% ΔSA.

Cell lysate from untreated, stretched (12% and 37% ΔSA) monolayers was examined for phospohorylated and total JNK, ERK, and p38. No alteration in MAPk phosphorylation from unstretched levels was observed at any time point in monolayers stretched to 12% ΔSA (Figure 1). Following 10 minutes of stretch to 37%ΔSA, phosphorylation of all MAPks was significantly increased compared to unstretched levels (Figure 1,*). By 30 minutes of cyclic stretch, phosphorylation of JNK and p38 had decreased such that they were no longer significantly more phosphorylated than unstretched levels, however ERK phosphorylation remained significantly elevated compared to unstretched controls. Post hoc analysis showed that phosphorylation of JNK was significantly higher following 10 minutes of stretch than following 60 minutes of stretch. Furthermore, following 10 minutes of stretch, phosphorylation levels of JNK and ERK were significantly higher than that of p38, and therefore we focus on the JNK and ERK pathways in our subsequent studies.

### SP600125 and U0126 inhibit stretch-induced phosphorylation of JNK and ERK respectively

**Figure 2:**
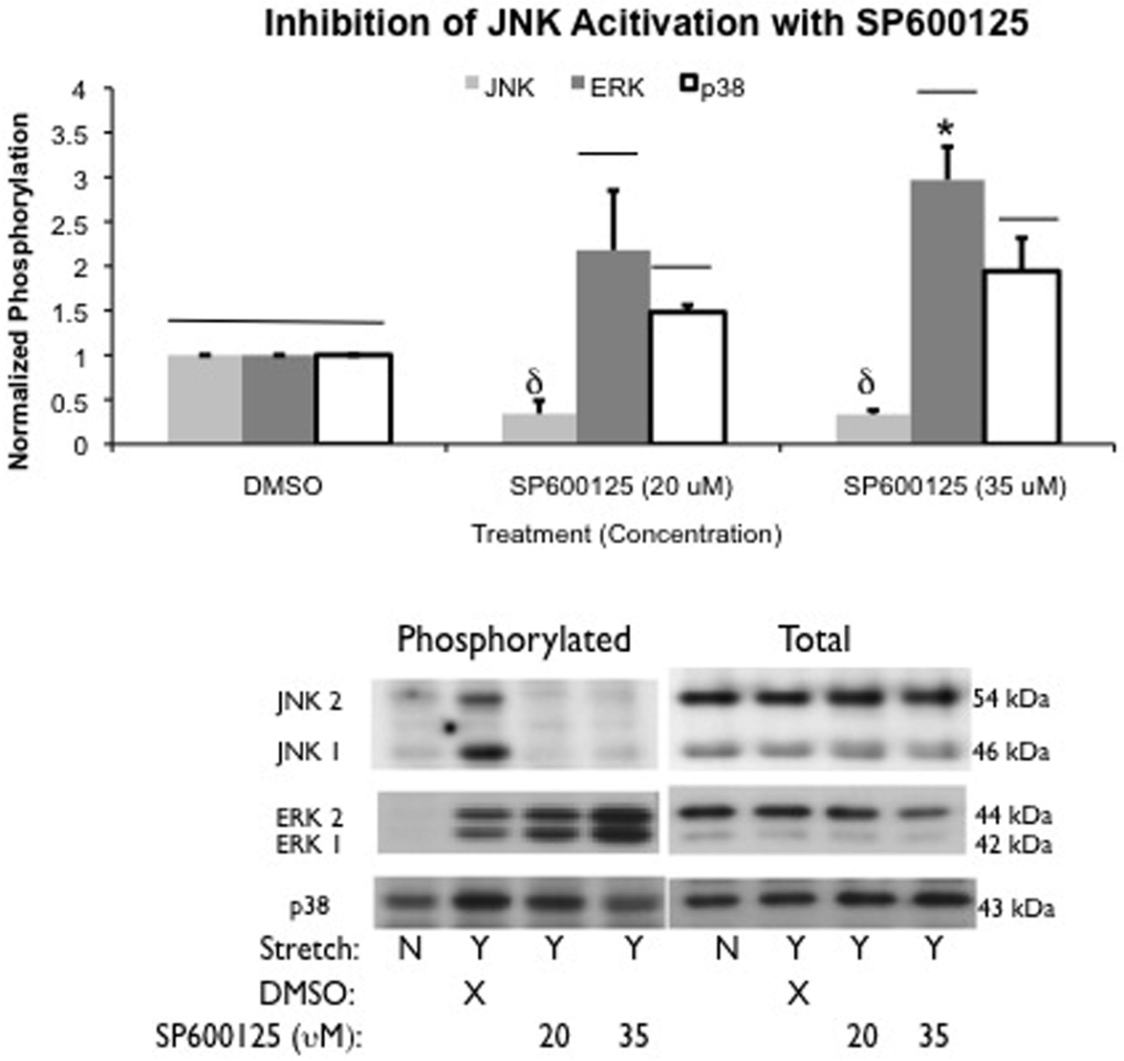
SP600125 inhibits JNK phosphorylation. Phosphorylation levels of JNK, ERK, and p38 MAPk following treatment with the inhibitor SP600125 (20 or 35 µM) or the control DMSO and 10 minutes of cyclic stretch at a rate of 0.25 Hz to a peak magnitude of 37% ΔSA normalized to stretched DMSO controls. Phosphorylation levels of all MAPks significantly increased above unstretched levels in DMSO treated wells. Both concentrations of SP600125 eliminated significant increases in JNK above unstretched levels. Stretch with a treatment of 35 µM of SP600125 resulted in significantly increased ERK phosphorylation compared to DMSO treated stretched wells. Significance is defined as p<0.05, and is depicted with (—) compared to unstretched, and (δ, lower) (*, higher) compared to DMSO treated. Representative western blots of phosphorylated and total MAPks with each treatment are also presented.

Pretreatment with 20 and 35 µM of the JNK inhibitor SP600125 significantly reduced the stretch-induced phosphorylation of JNK at 10 minutes compared to pretreatment with vehicle (DMSO) (Figure 2, δ). There was no significant difference in phosphorylation of JNK following treatment with larger or smaller concentrations of inhibitor. Use of SP600125 enhanced the stretch-induced phosphorylation of ERK (only at 35 µM) significantly compared to DMSO treated monolayers (Figure 2, *).

**Figure 3:**
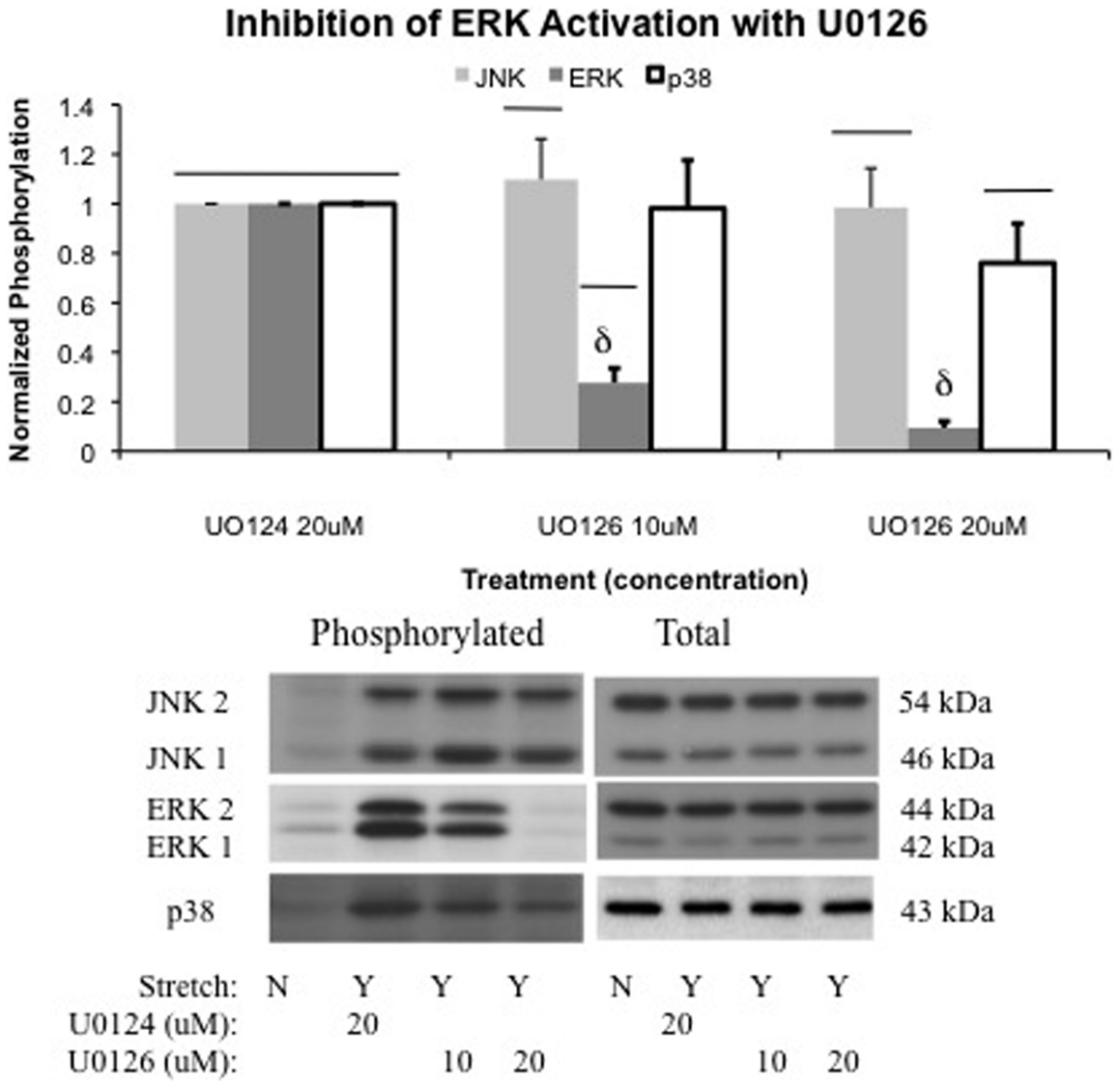
U0126 inhibits ERK phosphorylation. Phosphorylation levels of JNK, ERK, and p38 MAPk following treatment with the inhibitor U0126 (10 or 20 µM) or the control U0124 (20 µM) and 10 minutes of cyclic stretch at a rate of 0.25 Hz to a peak magnitude of 37% ΔSA normalized to stretched U0124 controls. Phosphorylation levels of all MAPks significantly increased above unstretched levels in U0124 treated wells. Following treatment with 10 or 20 µM of U0126 and 10 minutes of stretch, ERK phosphorylation was significantly reduced compared with U0124 treatment and stretch. Only treatment with 20 µM of U0126 eliminated significant increases in ERK above unstretched levels. Significance is defined as p<0.05, and is depicted with (—) compared to unstretched, and (δ) compared to U0124 treated. Representative western blots of phosphorylated and total MAPks with each treatment are also presented.

We also examined the effect of the ERK inhibitor U0126 (10 and 20 µM) on stretch-induced MAPk phosphorylation. Treatment with either concentration of the inhibitor reduced phosphorylation levels of ERK following stretch to 37% ΔSA for 10 minutes compared to negative control (U0124) treatments (Figure 3, δ). However, only 20 µM of U0126 prevented significant increases in phosphorylation of ERK compared to unstretched levels. The reduction in phosphorylation was not significantly different between inhibitor doses. Use of U0126, even at 20 µM, did not affect stretch-induced phosphorylation of p38 or JNK.

To ensure that use of the inhibitors (at the highest concentration used in the dose response studies) did not increase mortality rates in unstretched and stretched conditions, we treated separate wells with 35 µM SP600125, DMSO vehicle, 20 µM U0126 or 20 µM U0124, and either stretched (37% ΔSA, 0.25 Hz, 60 min) the cells or left them unstretched. In unstretched wells, the percent cell death was 0.51±0.27% for DMSO, 0.43±0.42 for SP600125 and 0.57±0.74 for U0126 treated wells. Toxicity of U0124, an inactive form of the inhibitor U0126, was not determined in unstretched wells. Following stretch, percent cell death of DMSO and U0124 treated wells (vehicle and negative control) was 9.04±3.71% and 11.08±4.37% respectively, while wells treated with SP600125 and U0126 had 9.41±5.59% and 10.19±5.20% cell death respectively. These data are consistent with our previously reported values of 0.3±0.5% in unstretched naïve controls and 9.2±4.4% in similarly stretched wells (37% ΔSA, 0.25 Hz 60 min), and show that neither treatment nor vehicle control altered cell viability under our experimental conditions [22].

### Inhibition of JNK not ERK Phosphorylation prevents barrier dysfunction following 60 minutes of cyclic stretch

**Figure 4:**
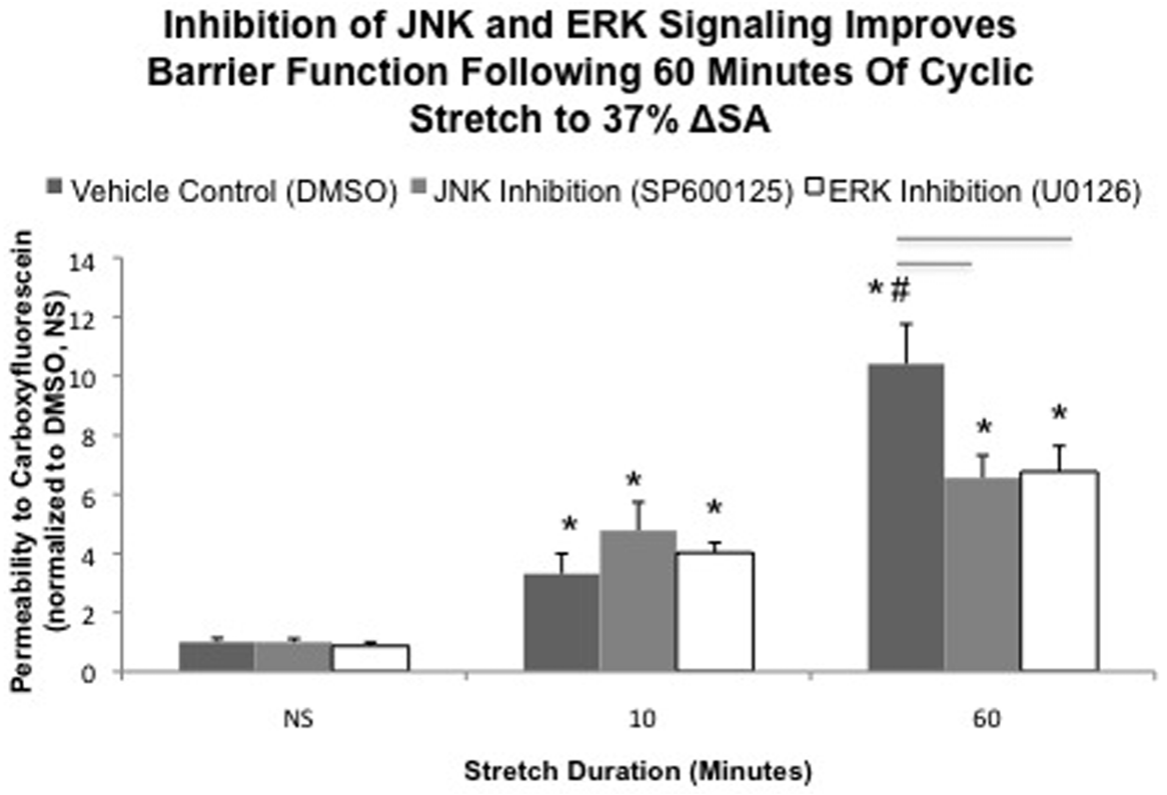
MAPk inhibition prevents stretch induced permeability increases. Permeability of monolayers treated with 35 µM of SP600125 or 20 µM of U0126 following 0, 10, or 60 minutes of stretch. Permeability of all monolayers stretched for 10 or 60 minutes significantly increased compared to unstretched monolayers (*). 60 minutes of stretch resulted in further increases above 10 minute levels in DMSO monolayers (#). Treatment with either SP600125 or U0126 prevented these further increases, and permeability values were significantly lower then in DMSO monolayers stretched for 60 minutes (-). Significance is defined as p<0.05.

As we reported previously, cyclic (0.25 Hz) mechanical stretch to 37% ΔSA for 60 minutes in our control groups resulted in significant increases in untreated monolayer permeability [4]. Our new data demonstrate significant permeability increases above unstretched levels after only 10 minutes of stretch, followed by a rapid deterioration of monolayer barrier function (Figure 4, dark gray bars).

Pre-treatment and stretch in the presence of either SP600125 (JNK inhibitor, Figure 4, light gray bars) or U0126 (ERK inhibitor, Figure 4, white bars) did not influence stretch-induced permeability increases following 10 minutes of cyclic stretch (37% ΔSA 0.25Hz), but the inhibitors significantly improved barrier properties at 60 minutes of cyclic stretch compared to untreated, stretched monolayers (Figure 4). Specifically, in control wells treated with DMSO and stretched, permeability increased by 10 minutes, and still further by 60 minutes. Treatment with SP600125 or U0126 abolished the long term increases in permeability.

### Reductions in Occludin Concentration Following Cyclic Stretch is Prevented by JNK Inhibition

**Figure 5:**
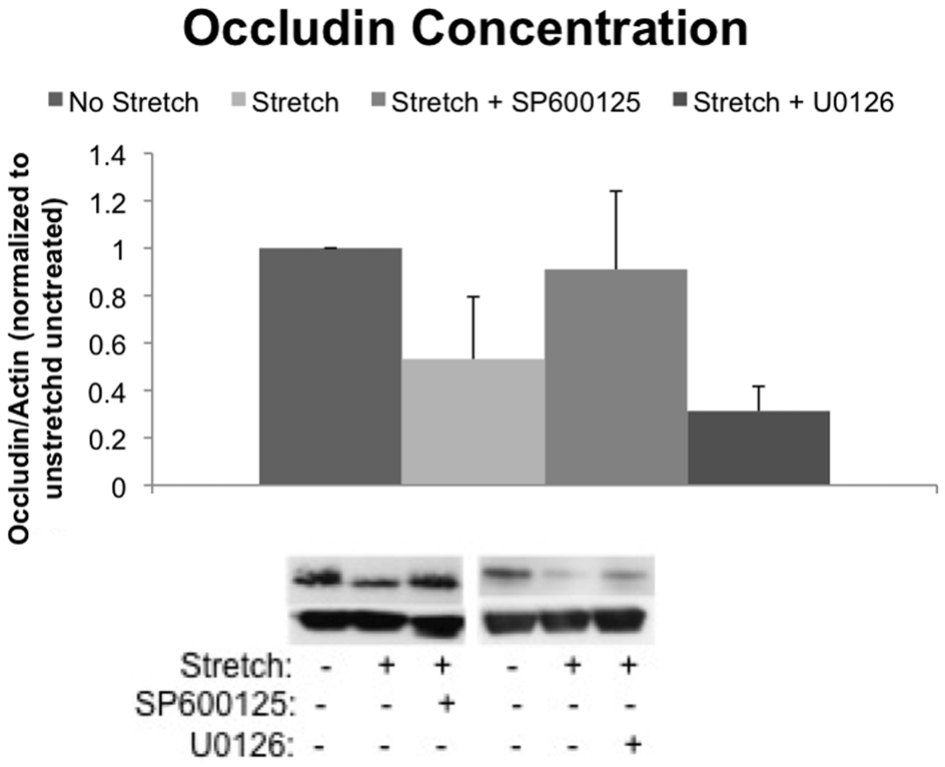
JNK inhibition prevents loss of occludin. Occludin concentration in cell monolayers significantly drops following 60 minutes of stretch (0.25 Hz, 37% ΔSA) compared to unstretched monolayers (*, p<0.05). Treatment with the JNK inhibitor SP600125 blocked occludin decreases following 60 minutes of stretch, however ERK inhibition with U0126 did not. (N≥3, μ ± SE).

Occludin concentration was analyzed before and after stretch as a marker of TJ integrity (Figure 5). As observed in a previous study, the concentration of occludin was unchanged following 1 hour of low magnitude (12% ΔSA) stretch, but dropped significantly (53% of control value) following 1 hour of high magnitude (37% ΔSA) stretch compared to unstretched controls [25]. Treatment with the JNK inhibitor SP600125 did not affect unstretched levels of occludin (data not shown), however JNK inhibition maintained occludin at 91% of control, thus preventing the stretch associated reductions of occludin. Inhibition of ERK did not prevent loss of occludin following 60 minutes of stretch.

**Figure 6:**
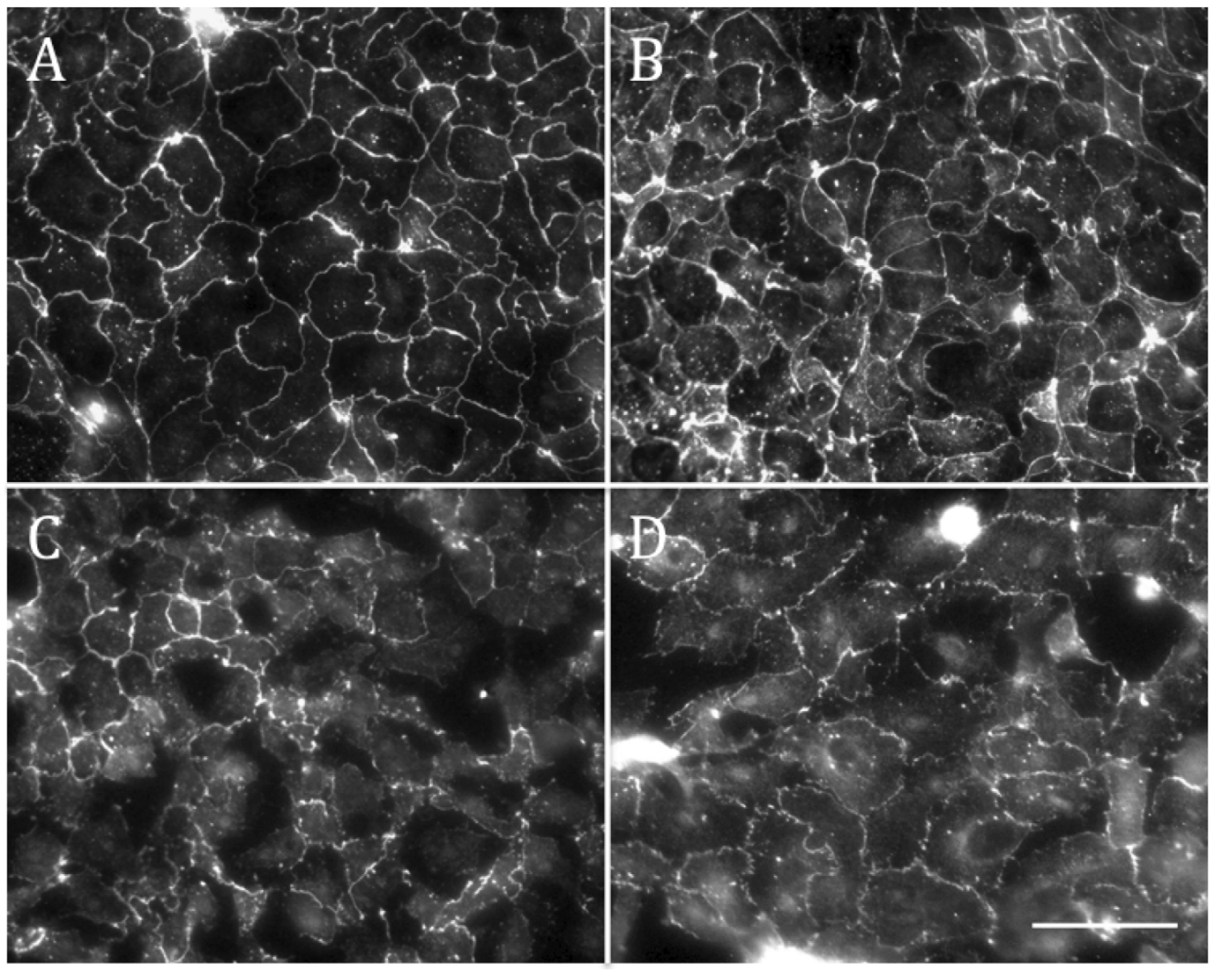
Occludin localization. Images of epithelial monolayers, stained with occludin antibodies, either treated with DMSO control (A and C) or SP600125 (B and D). Unstretched monolayers (A and B), exhibit strong staining of Occludin at the cell-cell junction. Following stretch, junctional staining is significantly reduced, and large holes form in the monolayer. SP600125 treatment reduces these alterations following stretch, however hole formation is still evident. (Scale bar 100 µm).

Furthermore, immunofluorescent images of occludin reveal alterations in TJ organization following stretch. Unstretched monolayers displayed a classic “chicken-wire” pattern of occludin at cell-cell junctions (Figure 6). Following 1 hour of stretch, junctional staining of occludin decreased, and large unstained intracellular regions appeared in the monolayer. Treatment with SP600125 did not alter the tight junction protein distribution in unstretched cells. Furthermore, stretch in the presence of SP600125 resulted in a reduction in the gap size and number, consistent with reduced epithelial permeability compared to the stretched untreated wells.

## Discussion

Previous *in vitro* studies have shown that stretch of cultured alveolar epithelial cells to a peak magnitude corresponding to 100% TLC results in increases in paracellular permeability [4], [21]. In the current study, we explore the MAPk phosphorylation as a possible mechanism underlying stretch-induced epithelial barrier dysfunction, because others have shown these pathways to regulate the formation of ion resistant tight junctions in mouse mammary epithelial cells, and wound healing in primary human epithelial cells [26], [27].

As previously described, we isolated and cultured alveolar epithelial cells for 5 days until confluent type I like monolayers were formed, at which point the cells were cyclically (0.25 Hz) stretched for 10, 30, or 60 minutes to a magnitude of 12% or 37% ΔSA, which corresponds to 70% or 100% TLC. We have shown, using this model of the alveolar epithelium, that 60 minutes of stretch to 37% ΔSA and this rate produces significant increases in epithelial permeability. We analyzed phosphorylation levels of JNK, ERK and p38 MAP kinases in either unstretched monolayers, or those stretched to each magnitude, and found significant phosphorylation (JNK, ERK, and p38) above unstretched only in the high magnitude group. Phosphorylation was increased by 10 minutes of stretch and subsided with increased stretch duration. By 60 minutes only ERK phosphorylation was significantly elevated compared with unstretched controls.

Pretreatment of the monolayers with specific inhibitors of JNK (SP600125) or ERK (U0126) prevented stretched induced phosphorylation of each MAPk respectively. We did not inhibit p38, and therefore cannot comment on any potential role it plays in barrier dysfunction, as increases in p38 phosphorylation observed at 10 minutes were significantly less than that of JNK and ERK, rapidly subsided, and were not significantly greater than phosphorylation levels at 30 or 60 minutes which were no different than unstretched levels. We assessed barrier function of monolayers stretched in the presence of the inhibitors, and found that neither inhibitor had any effect on permeability following 10 minutes of stretch, but after 60 minutes of stretch, both JNK and ERK inhibition significantly attenuated further permeability increases.

It is possible that the beneficial effects of JNK inhibition are due to an upregulation of the ERK signaling pathway, as ERK has been linked to cell differentiation and enhanced cell survival following a stress event [11], [28]. Activation of JNK has been shown to reduce ERK phosphorylation by inhibiting activation of upstream kinases, and in fact we observe increases in ERK phosphorylation following stretch in the presence of the JNK inhibitor SP600125 [29]. However, ERK inhibition with U0126 also significantly reduced stretch induced permeability, therefore, we cannot conclude that SP600125 rescues permeability due to its effect on ERK phosphorylation.

Initially, we hypothesized that the beneficial effect of inhibition JNK phosphorylation, not ERK phosphorylation, could be due to inhibition of apoptosis. Historically, activation of the JNK and ERK signaling pathways have been shown to produce pro-apoptotic and anti-apoptotic cellular reactions, respectively [30]. Therefore, we postulated that loss of barrier function could partially be due to a reduction in cell death. However viability in stretched wells was unaffected by either inhibitor compared to their vehicle controls. Therefore we postulated that modulation of epithelial permeability was due to alterations in the cell-cell junctions rather than loss of monolayer integrity due to cell death.

Activation of JNK signaling has been associated with phosphorylation of tight junction proteins ZO-1 and occludin [31]. Increased phosphorylation of these proteins could lead to reorganization of the tight junction complex, and increases in epithelial permeability. Data from, Chen *et. al.* demonstrates that blocking TNFα activation of JNK inhibited ZO-1 removal from the tight junction complex and preserved barrier function in endothelial cells [32]. The current, as well as previous, studies show that occludin content drops significantly following an hour of stretch, and that occludin is redistributed throughout the cell at this time point [25]. Inhibition of JNK signaling prevents drops in occludin concentration, while reducing the extent to which occludin is redistributed. These findings support the hypothesis that JNK signaling promotes junctional disassembly.

We had further hypothesized that inhibition of an alternative MAPk signaling pathway, ERK, would similarly prevent permeability increases following stretch, also by reducing the extent of occludin degradation. This hypothesis was based on previous studies that demonstrate ERK phosphorylation regulates tight junction disassembly following cigarette smoke damage of human cell lines and TGFβ induced epithelial mesenchymal transition in murine epithelial cells [33], [34]. Contradicting data does exist showing a protective affect of epidermal growth factor (EGF) induced ERK phosphorylation on occludin expression [35]. In our study we observe inhibition of ERK signaling prevents increases in permeability, however no effect on occludin was observed. Alternative mechanisms of ERK induced barrier dysfunction could include modulation of calcium dependent calpain activation and degradation of cellular adhesions [36], [37]. We have previously shown permeability increases to be dependant on calcium signaling and actin remodeling, therefore future studies will explore the link between stretch induced ERK activation and these alternative mechanisms of epithelial barrier dysfunction [4].

In summary, we stretched cultured alveolar epithelial cells to a magnitude shown to produce significant increases in permeability, and explored MAPk signaling as a possible mechanism of these increases. We found that JNK, ERK, and p38 MAP kinases were significantly phosphorylated by stretch. Furthermore, large magnitude stretch for 60 minutes increased monolayer permeability, produced large gaps in the monolayer, and reduced expression of the tight junction protein occludin. Inhibition of either JNK or ERK reduced the stretch-induced monolayer permeability, but inhibition of JNK also reduced alterations in occludin concentration and cellular localization. We conclude that MAPk signaling has the potential to modulate stretch-induced alveolar epithelial injury, and propose further studies should be conducted to identify opportunities for reducing tight junction disruption.
